# On-line monitoring of the live cell concentration in bioreactors based on a rocking platform

**DOI:** 10.1186/1753-6561-5-S8-P125

**Published:** 2011-11-22

**Authors:** John Carvell, Matt Lee

**Affiliations:** 1 Aber Instruments Ltd., Aberystwyth, SY23 3AH, UK

## Background

Aber Instruments first introduced the concept of using disposable live-biomass probes in 2009 [1]. The probe has been carefully designed to be welded into most single use bioreactors and is suitable for bags with agitators *eg* the Hyclone SUB and the Sartorius Stedim Biostat Cultibag STR, or those using the rocker type platform eg Sartorius Stedim Biostat Cultibag RM and GE Wave bioreactors . The disposable biomass probe has four electrodes with the same dimensions as the existing reusable production biomass probes with flush platinum electrodes that are used in cGMP manufacture worldwide. The electrode support material is HDPE that meets FDA and USP Class VI requirements and the probe can withstand gamma sterilisation and be stored for prolonged periods before use. The disposable probe is easily connected to a mini-lightweight Futura pre-amplifier so that the weight load or torque on the bag is minimal and the bulk of the electronics is then located well away from the bag. The disposable probe has already been welded into different bags including Sartorius Stedim Biotech CultiBag RM bioreactors.

## Materials and methods

Experiments were performed with a Futura MRF Mini-Remote Futura (Aber Instruments Ltd) and 58mm diameter disposable “Coupon” probes. The probes were welded into the RM Cultibags by Sartorius Stedim. The tests performed with SF9 cells at GSK used a combination of the Biomass Monitor 220 (Aber Instruments, UK) and a Futura MRF Mini-Remote Futura pre-amplifier. The media used was Excell 420 from SAFC Biosciences.

### Tests with Rocking Motion bags

One of the challenges of installing any on-line probe in a rocking motion bioreactor is that the sensor will be exposed to varying levels of fluid. When the bag is used at the minimum recommended volume, the probe will also be exposed momentarily to the gaseous headspace of the bioreactor. In the very first tests with the disposable probe installed in a 5L Sartorius Stedim Biotech CultiBag RM filled with just media, the unfiltered capacitance and conductivity profiles were shown to become increasingly noisier as the speed of the rocking action was increased.

An advanced rocker filter algorithm, including an "anti-beat" mechanism, was developed to work automatically at varying rocker speeds such that the Futura does not have to monitor the rocking speed or synchronise the readings to the angle of the rocking table. Further experiments were then performed with disposable probes mounted within a simulated bag platform on a rocker set at a higher than average speed for cell culture applications (47rpm). The volume of media (500ml) was set to a maximum depth of 20mm at full tilt and the minimum depth over the electrodes was 1mm during the rocking process.

The resulting capacitance and conductivity traces showed that a normal average filter flattened out the noise, however, if only the averaging filter is used, the output filtered value was markedly lower than the steady state value. The new rocker algorithm successfully filtered out the noise caused by the rocking motion and was far better at maintaining the steady state value.

**Figure 1:**
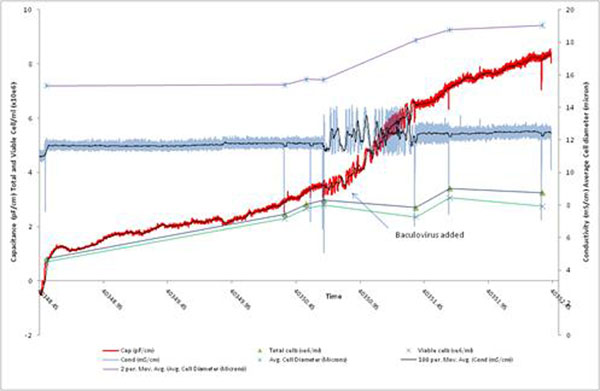
Capacitance and conductivity measurements in a 50-L Sartorius (25 L working volume) rocking-motion CultiBag system (R. Tanner, GlaxoSmithKline, UK)

The disposable probe also has been assessed with a prototype 50-L Sartorius Stedim Biotech CultiBag RM (25L working volume) in monitoring the growth of Sf9 insect cells (Figure 1). The probe tracked viable cell density before addition of a baculovirus for transient recombinant protein expression. The biomass probe successfully detected a valid infection by showing a rapid increase in signal caused by increasing volumes of the infected cells.

## Conclusions

Use of RF impedance to monitor cell culture processes is well established in biopharmaceutical applications. Introduction of the Futura biomass monitor allows this technology to be used with confidence on rocking motion disposable bioreactors, from process development through cGMP production.
